# Elimination of Specific miRNAs by Naked 14-nt sgRNAs

**DOI:** 10.1371/journal.pone.0038496

**Published:** 2012-06-04

**Authors:** Masayuki Takahashi, Reyad A. Elbarbary, Mayumi Abe, Mari Sato, Tetsuo Yoshida, Yoji Yamada, Masato Tamura, Masayuki Nashimoto

**Affiliations:** 1 Department of Applied Life Sciences, Niigata University of Pharmacy and Applied Life Sciences, Niigata, Japan; 2 Department of Biochemistry and Molecular Biology, Hokkaido University Graduate School of Dental Medicine, Sapporo, Japan; 3 Biologics Research Laboratories, Kyowa Hakko Kirin Company Limited, Machida, Japan; IPMC, CNRS UMR 6097 UNSA, France

## Abstract

tRNase Z^L^-utilizing efficacious gene silencing (TRUE gene silencing) is a newly developed technology to suppress mammalian gene expression. TRUE gene silencing works on the basis of a unique enzymatic property of mammalian tRNase Z^L^, which is that it can recognize a pre-tRNA-like or micro-pre-tRNA-like complex formed between target RNA and artificial small guide RNA (sgRNA) and can cleave any target RNA at any desired site. There are four types of sgRNA, 5′-half-tRNA, RNA heptamer, hook RNA, and ∼14-nt linear RNA. Here we show that a 14-nt linear-type sgRNA against human miR-16 can guide tRNase Z^L^ cleavage of miR-16 *in vitro* and can downregulate the miR-16 level in HEK293 cells. We also demonstrate that the 14-nt sgRNA can be efficiently taken up without any transfection reagents by living cells and can exist stably in there for at least 24 hours. The naked 14-nt sgRNA significantly reduced the miR-16 level in HEK293 and HL60 cells. Three other naked 14-nt sgRNAs against miR-142-3p, miR-206, and miR-19a/b are also shown to downregulate the respective miRNA levels in various mammalian cell lines. Our observations suggest that in general we can eliminate a specific cellular miRNA at least by ∼50% by using a naked 14-nt sgRNA on the basis of TRUE gene silencing.

## Introduction

tRNase Z^L^ is a long form of tRNA 3′ processing endoribonuclease (tRNase Z, or 3′ tRNase) [1], [2]. In human cells, nuclear full-length tRNase Z^L^ works on precursor tRNAs to process them by removing their 3′ trailer, whereas cytosolic Δ30 tRNase Z^L^ appears to work on mRNAs to modulate gene expressions by cleaving them under the direction of cellular small noncoding RNAs such as 5′-half-tRNA and miRNA [3], [4]. It has been shown that cytosolic tRNase Z^L^ modulates the PPM1F gene expression by cleaving its mRNA under the direction of 5′-half-tRNA^Glu^ [3] and that miR-103 can downregulate gene expression through directing mRNA cleavage by cytosolic tRNase Z^L^ [4]. The cytosolic tRNase Z^L^ together with the small noncoding RNAs appears to form a broad gene regulatory network.

We have developed a technology for suppressing the expression of a target gene by modulating this gene regulatory network under the aegis of artificial small guide RNA (sgRNA) [5]–[9]. This technology works on the basis of a unique enzymatic property of mammalian tRNase Z^L^, which is that it can recognize a pre-tRNA-like or micro-pre-tRNA-like complex formed between target RNA and artificial sgRNA and can cleave any target RNA at any desired site [10]–[16]. There are four types of sgRNA, 5′-half-tRNA [11], RNA heptamer [12], hook RNA [15], and ∼14-nt linear RNA [16], and this technology is termed TRUE gene silencing after tRNase Z^L^-utilizing efficacious gene silencing. The efficacy of TRUE gene silencing can become comparable to that of the RNA interference [7] and can surpass it in some cases [8].

miRNAs play important regulatory roles in many cellular processes, and their dysfunction appears to cause many diseases including cancer and heart disease [17]. It has been shown in human cells that miR-15a and miR-16 work as tumor suppressors, while miR-17-92, miR-19a/b, miR-21, and miR-181a/b are oncogenic [18]–[20]. miR-122, which is abundant in liver cells, has been shown to be a regulator of fatty-acid metabolism [21], and its expression appears to be essential for replication of hepatitis C virus [22]. Thus we can expect that elimination of specific miRNAs in the cells would lead to cures of some diseases. Indeed, downregulation of the miR-19 or miR-181 level in myeloma cells by an antisense nucleic acid has been demonstrated to suppress tumor growth in nude mice [20]. And oligonucleotides against miR-122 can reduce a blood cholesterol level [21] and can also suppress the replication of hepatitis C virus [22].

In this paper, we investigated if TRUE gene silencing can eliminate specific miRNAs from human cells. And we show that human tRNase Z^L^ can cleave miRNA under the direction of 14-nt linear-type sgRNA *in vitro* and that naked 14-nt sgRNA can significantly downregulate miRNA expression *in vivo*.

## Materials and Methods

### RNA/DNA Synthesis

The following eleven 5′- and/or 3′-phosphorylated RNAs with full 2′-*O*-methyl modifications, 3′-Alexa-568-labeled 5′-phosphorylated sgR16(1–14) with full 2′-*O*-methyl modifications, 3′-FITC-labeled 5′-phosphorylated sgRNA14 with full 2′-*O*-methyl modifications, and 5′-FITC-labeled miR-16 were chemically synthesized by Nippon Bioservice: sgR16(1–14), 5′-pUUUACGUGCUGCUA(p)-3′; sgR16(9–22), 5′-pCGCCAAUAUUUACG-3′; sgR16(1–22), 5′-pCGCCAAUAUUUACGUGCUGCUA(p)-3′; sgR16(1–12), 5′-pUACGUGCUGCUA-3′; sgR16(6–17), 5′-pAUAUUUACGUGC-3′; sgR16(4–17), 5′-pAUAUUUACGUGCUG-3′; sgR16(4–15), 5′-pAUUUACGUGCUG-3′; sgR142(1–14), 5′-pUAGGAAACACUACAp-3′; sgR142(1–23), 5′-pUCCAUAAAGUAGGAAACACUACAp-3′; sgR206(1–14), 5′-pUUCCUUACAUUCCAp-3′; sgR19(1–14), 5′-pCAUGGAUUUGCACAp-3′; sgRNA14, 5′-pGGGGGCGGCCCCCG-3′; miR-16, 5′-UAGCAGCACGUAAAUAUUGGCG-3′.

The following DNA probes and primers were obtained from Hokkaido System Science: miR-16 probe, 5′-CGCCAATATTTACGTGCTGCTA-3′; 5S rRNA probe, 5′-AAGCCTACAGCACCCGGTATT-3′; miR-16 reverse transcription primer, 5′-GTCGTATCCAGTGCAGGGTCCGAGGTATTCGCACTGGATACGACCGCCAA -3′; miR-16 forward primer, 5′-CGCGCTAGCAGCACGTAAAT-3′; miR-16 and miR-19a/b reverse primer, 5′-GTGCAGGGTCCGAGGT-3′; miR-19a/b reverse transcription primer, 5′-GTCGTATCCAGTGCAGGGTCCGAGGTATTCGCACTGGATACGACTCAGTT-3′; miR-19a/b forward primer, 5′-CGCGCTGTGCAAATCTATGC-3′; 5S rRNA forward primer, 5′-GTCTACGGCCATACCACCCTG-3′; 5S rRNA reverse primer, 5′-AAGCCTACAGCACCCGGTATT-3′; Bcl-2 mRNA forward primer, 5′-GCCCTCACTGGCCTCCTCCA-3′; Bcl-2 mRNA reverse primer, 5′-GTGACAGGCCCAGCCACACC-3′; β-actin mRNA forward primer, 5′-CTGGAACGGTGAAGGTGACA-3′; β-actin mRNA reverse primer, 5′-AAGGGACTTCCTGTAACAACGCA-3′. The siRNA targeting the human tRNase Z^L^ mRNA was obtained from Qiagen: sense, r(GACUCCGAGUCGAAUGAAA)d(TT); antisense, r(UUUCAUUCGACUCGGAGUC)d(TG). Silencer Negative Control No. 1 siRNA (#4611; Ambion) was used as a scramble siRNA.

### *In vitro* RNA Cleavage Assay

*In vitro* RNA cleavage assays for the FITC-labeled miR-16 (2 pmol) were carried out at 37°C in the presence of the unlabeled sgRNAs (20 pmol) using histidine-tagged human Δ30 tRNase Z^L^ (50 ng) in a mixture (6 µl) containing 10 mM Tris-HCl (pH 7.5), and 3.3 mM MgCl_2_. After resolution of the reaction products on a 15% polyacrylamide-8 M urea gel, the gel was analyzed with a Typhoon 9210 (GE Healthcare).

### Cell Culture and Transfection

Various mammalian cells, HEK293 [23], HeLa [24], Jurkat [25], HL60 [26], DAUDI (obtained from RIKEN BioResource Center, Tsukuba, Japan), C2C12 [27], and RPMI-8226 [28], were cultured in RPMI-1640 or DME media (Wako) supplemented with 10% fetal bovine serum (FBS; MP Biomedicals) and 1% penicillin-streptomycin (Invitrogen) at 37°C in 5% CO_2_ humidified incubator.

HEK293 cells were transfected with sgRNA or with sgRNA and scramble or anti-tRNase-Z^L^ siRNA using Lipofectamine 2000 (Invitrogen) according to the manufacturer’s protocol, and cultured for further 42 hours.

### Northern Analysis

Total RNA was extracted with ISOGEN. The RNA samples (10 µg) were separated by 15% polyacrylamide/8 M urea gel electrophoresis, and electrically transferred to a Hybond N^+^ membrane (GE Healthcare). The membrane was ultraviolet-crosslinked, probed with a 5′-^32^P-labeled deoxyoligonucleotide in a QuickHyb buffer (Stratagene) at 45°C, and analyzed with the Typhoon 9210.

### Real-Time PCR

Total RNA was extracted from cells using ISOGEN. The cellular amounts of miR-16, miR-142-3p, and miR-206 were quantitated by a StepOne Real Time PCR System using TaqMan MicroRNA Assays and TaqMan MicroRNA Reverse Transcription Kit (Applied Biosystems). The miR-16, miR-19a/b, Bcl-2 mRNA and β-actin mRNA levels were quantitated by real-time PCR using a LightCycler 480 SYBR Green I Kit (Roche).

### Western Analysis

Whole cell extracts dissolved in a buffer (50 mM Tris-HCl pH 6.8, 2% SDS, 10% glycerol, 100 mM dithiothreitol) were separated by SDS/7.5% polyacrylamide gel electrophoresis, and transferred to a nitrocellulose membrane. The membrane was probed with polyclonal antibodies raised to a human tRNase Z^L^ peptide (amino acid 812–826) or a monoclonal antibody against human β-actin (Sigma) using the ECL Western Blotting Detection System (GE Healthcare).

### Fluorescent Microscopic Analysis

HL60 and RPMI-8226 cells (5×10^4^ cells/well) were cultured in 24-well plates in the absence and presence of 1 µM Alexa-568-labeled sgR16(1–14), and HEK293 cells (5×10^4^ cells) were likewise in a fibronectin-coated chamber slide. After 24 hours, the cells were rinsed twice with 1×phosphate-buffered saline (PBS) and fixed with 4% paraformaldehyde in 1×PBS for 3 min. Hoechst 33342 (DOJINDO) was used to stain DNA, and the cells were analyzed with the fluorescent microscope system Axio Imager.M2 (Zeiss).

### sgRNA Survivability Test

Human cells (0.8–1×10^5^ cells/well) were cultured in a 24-well plate, and 24 hours after the addition of 1 µM of the 3′-Alexa-568-labeled sgR16(1–14) or the 3′-FITC-labeled sgRNA14 to the medium, the cells were harvested and washed four times with 1 ml of 1×PBS. Total RNA was extracted from the cells with ISOGEN, and separated on a 20% polyacrylamide-8 M urea gel. The gel was analyzed with the Typhoon 9210.

### Statistical Analysis

Differences between control and experimental groups were evaluated by the Student’s *t*-test.

## Results and Discussion

### tRNase Z^L^ can Cleave miR-16 under the Direction of a 14-nt sgRNA

**Figure 1:**
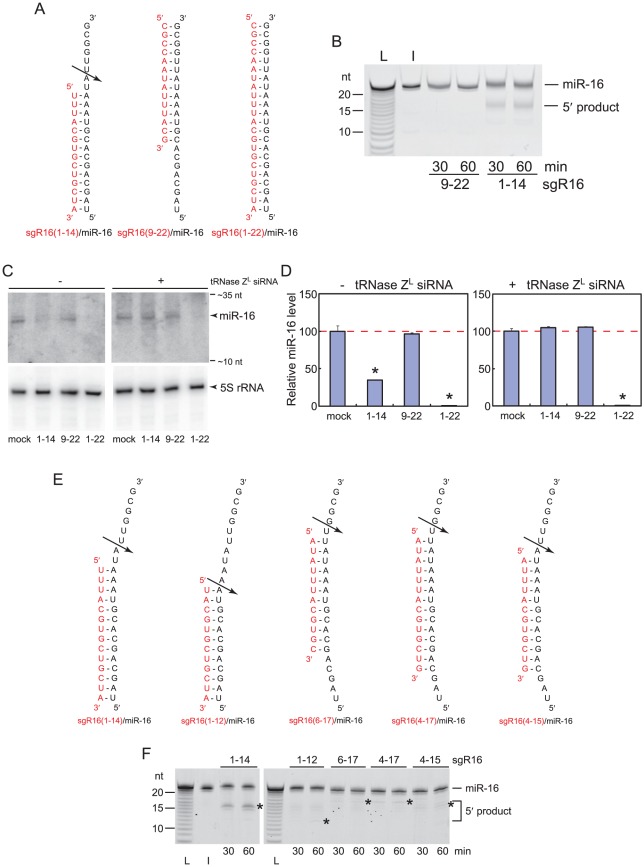
Downregulation of the miR-16 expression by TRUE gene silencing. (A and E) Structures of the complexes of sgRNAs with human miR-16. An arrow indicates the tRNase Z^L^ cleavage site. (B and F) *In vitro* tRNase Z^L^ cleavage assays. 5′-fluorescein-labeled miR-16 was incubated with recombinant human Δ30 tRNase Z^L^ in the absence or presence of sgRNA, which was phosphorylated at the 5′ end but not at the 3′ end. The cleavage products were analyzed on a denaturing 15% polyacrylamide gel. L, alkaline ladder of miR-16; I, input RNA. (C) Northern analysis for human miR-16. Total RNA was extracted from the HEK293 cells that were transfected with mock, 100 nM of sgR16(1–14), sgR16(9–23) or sgR16(1–23) without (left) or together with 100 nM of the anti-tRNase-Z^L^ siRNA (right). sgR16(1–14), sgR16(9–23), and sgR16(1–23) were phosphorylated at the 5′ end but not at the 3′ end. The total RNA (10 µg) was separated on a denaturing 15% polyacrylamide gel. Bromophenol blue (∼10 nt) and xylene cyanol (∼35 nt) were used as size markers. (D) The same RNA samples as in C were analyzed by a StepOne Real Time PCR System. The miRNA levels are normalized against the RNU48 levels. Error bars indicate s.d. (n = 3). *, P<0.005.

To examine if tRNase Z^L^ can cleave miRNA under the direction of sgRNA, we performed an *in vitro* tRNase Z^L^ cleavage assay for human miR-16. A 14-nt linear-type sgRNA, sgR16(1–14), which is complementary to the 1st–14th nucleotides of miR-16, and a control 14-nt RNA, sgR16(9–22), which is complementary to 9th–22nd nucleotides of miR-16, were chemically synthesized as 2′-*O*-methyl RNA (Figure 1A). As expected, miR-16 was cleaved after the 16th A by tRNase Z^L^ under the direction of sgR16(1–14), but not sgR16(9–22) (Figure 1B).

We also tested 4 more linear-type sgRNAs against miR-16 for their guiding ability. These sgRNAs, sgR16(1–12), sgR16(6–17), sgR16(4–17), and sgR16(4–15), are designed to be complementary to 12 or 14 nucleotides of miR-16 (Figure 1E,F). All these sgRNAs guided tRNase Z^L^ cleavage of miR-16, albeit inefficiently compared with sgR16(1–14). The cleavages directed by sgR16(6–17), sgR16(4–17), and sgR16(4–15) occurred primarily after a nucleotide corresponding to the discriminator nucleotide, whereas those by sgR16(1–14) and sgR16(1–12) occurred primarily 1-nt downstream and 1-nt upstream, respectively. These cleavage site fluctuations have been previously described as a property of tRNase Z^L^ [16], [29].

These results suggest that both the length and the binding site of sgRNA are important for its efficient guiding ability. We focused on 14-nt sgRNAs that are complementary to the 1st–14th nucleotides of miRNAs for the following *in vivo* analyses.

### Downregulation of the miR-16 level by TRUE Gene Silencing

Next, we examined if sgR16(1–14) works as sgRNA against miR-16 in human cells. sgR16(1–14), sgR16(9–22), or the 22-nt antisense RNA sgR16(1–22) was transfected into HEK293 cells, and the cellular miR-16 level was analyzed by northern blotting and real-time PCR. sgR16(1–14) and sgR16(1–22) significantly downregulated the miR-16 level, whereas sgR16(9–22) did not at all (Figure 1C,D). We also carried out co-transfection experiments with siRNA against human tRNase Z^L^ mRNA. The reduction in the miR-16 level by sgR16(1–14) was restored by downregulating the tRNase Z^L^ expression, while the reduction by sgR16(1–22) was not affected (Figure 1C,D).

We performed similar experiments under slightly different conditions, in which 5′- and 3′-phosphorylated sgR16(1–14) and sgR16(1–22) were used, and also analyzed tRNase Z^L^ levels by western blotting. In one set of the experiments, we obtained results similar to the above ones, but restoration of the miR-16 level reduction by downregulating the tRNase Z^L^ expression was incomplete (Figure S1A). This may be due to incomplete elimination of tRNase Z^L^ (Figure S1B) and/or due to a possible weak binding and sequestering effect of sgR16(1–14).

**Figure 2:**
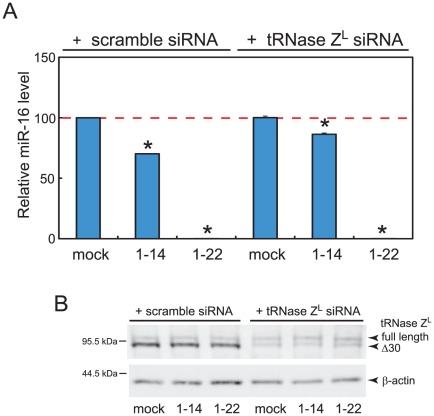
tRNase Z^L^ is responsible at least partly for the reduction in the miR-16 level by sgR16(1–14). (A) Quantitation of the miR-16 level in HEK293 cells with a LightCycler 480 SYBR Green I Kit. The HEK293 cells were transfected with mock, 35 nM of sgR16(1–14) or sgR16(1–22) together with 50 nM of the scramble siRNA or the anti-tRNase-Z^L^ siRNA. sgR16(1–14) and sgR16(1–22) were phosphorylated at both 5′ and 3′ ends. The miR-16 levels are normalized against the 5S rRNA levels. Error bars indicate s.d. (n = 3). *, P<0.001. (B) Western blot analysis. The tRNase Z^L^ level under the above transfection conditions was analyzed. β-actin was used as a loading control.

In another set of the experiments, in which a scramble siRNA was used as a control, we also obtained similar results (Figure 2). Together, these results suggest that tRNase Z^L^ is responsible at least partly for the reduction by sgR16(1–14) but not by sgR16(1–22) and that TRUE gene silencing can downregulate the miRNA expression. The mechanism by which sgR16(1–22) acts on the miR-16 level would be through binding and sequestering [20]–[22].

### Naked Linear-type sgRNA can be Taken up by Living Cells

**Figure 3:**
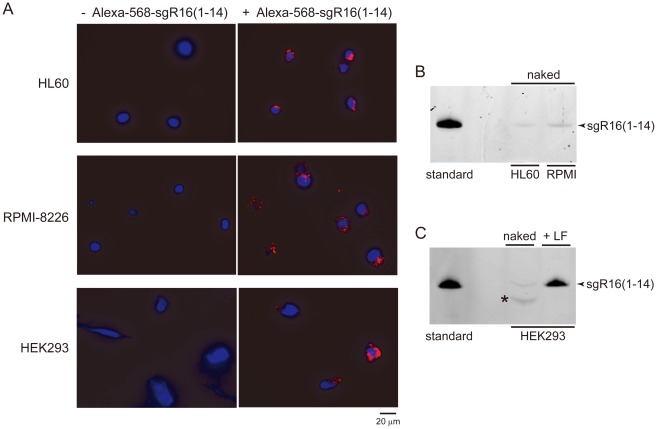
Naked linear-type sgRNA can be taken up by living cells. (A) Microscopic images of HL60, RPMI-8226 and HEK293 cells. The cells were fixed and stained with Hoechst 3342, 24 hours after the cells were cultured in the absence or presence of 1 µM of the naked 3′-Alexa-568-labeled sgR16(1–14). (B and C) sgRNA survivability tests. The 3′-Alexa-568-labeled sgR16(1–14) was analyzed on a denaturing 20% polyacrylamide gel. The sgRNA recovered from inside the cells are shown, which took it up nakedly or with the aid of Lipofectamine 2000 (LF). The band denoted by an asterisk would correspond to a degradation product of sgR16(1–14). Standard, 3′-Alexa-568-labeled sgR16(1–14).

We investigated how efficiently linear-type sgRNA can be taken up by living cells without any transfection reagents and can downregulate the miRNA expression. HL60 cells were cultured in the presence of 1 µM of the naked 14-nt 3′-Alexa-568-labeled sgR16(1–14) and analyzed with a fluorescent microscope. This sgRNA was indeed efficiently taken up without any transfection reagents by HL60 cells (Figure 3A). RPMI-8226 and HEK293 cells also took up the naked sgRNA efficiently (Figure 3A). We also showed that another 14-nt RNA, sgRNA14, can be taken up nakedly by HeLa, HEK293, and Jurkat cells (Figure S2A,B and Text S1).

Furthermore, we demonstrated by polyacrylamide gel analysis of total RNA from HL60, RPMI-8226, and HEK293 cells that at least a part of the taken-up sgR16(1–14) molecules exist stably for at least 24 hours in the cells (Figure 3B,C). And at least a part of sgRNA14 molecules were also stable for at least 24 hours in HeLa, HEK293, and Jurkat cells (Figure S2C). However, the amount of sgR16(1–14) recovered from HEK293 cells that took it up with the aid of a transfection reagent was much higher than that recovered from the cells that took it up nakedly (Figure 3C), suggesting that sgR16(1–14) can be taken up more easily and/or can exist more stably in the presence of the transfection reagent.

### Reduction in the miR-16 Level by a Naked 14-nt sgRNA

**Figure 4:**
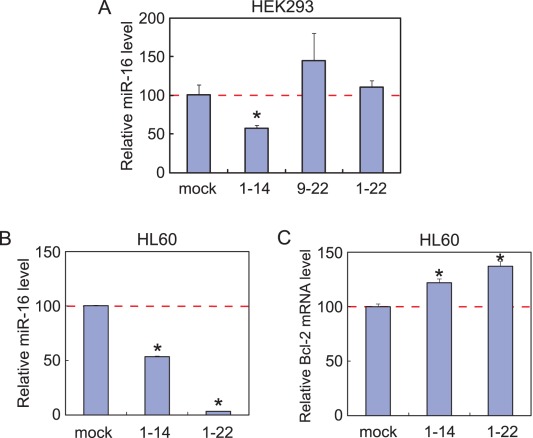
Reduction in the miR-16 level by a naked 14-nt sgRNA. (A) Quantitation of the miR-16 level in HEK293 cells with a StepOne Real Time PCR System. Total RNA was extracted from the HEK293 cells that were cultured for 24 hours in the absence or presence of sgR16(1–14), sgR16(9–22), or sgR16(1–22), which was phosphorylated at the 5′ end but not at the 3′ end. The miR-16 levels are normalized against the RNU48 levels. Error bars indicate s.d. (n = 3). *, P<0.05. (B) Quantitation of the miR-16 level in HL60 cells with a LightCycler 480 SYBR Green I Kit. Total RNA was extracted from the HL60 cells that were cultured for 24 hours in the absence or presence of sgR16(1–14) or sgR16(1–22). sgR16(1–14) and sgR16(1–22) were phosphorylated at both 5′ and 3′ ends. The miR-16 levels are normalized against the 5S rRNA levels. Error bars indicate s.d. (n = 3). *, P<0.001. (C) Real-time PCR analysis for the Bcl-2 mRNA level in HL60 cells. Total RNA was extracted from the HL60 cells that were cultured for 24 hours in the absence or presence of sgR16(1–14) or sgR16(1–22). sgR16(1–14) and sgR16(1–22) were phosphorylated at both 5′ and 3′ ends. The Bcl-2 mRNA levels are normalized against the β-actin mRNA levels. Error bars indicate s.d. (n = 3). *, P<0.001.

We cultured HEK293 cells in the presence of 1 µM of naked sgR16(1–14), sgR16(9–22), or sgR16(1–22), and analyzed the cellular miR-16 level by real-time PCR. These RNAs were phosphorylated at the 5′ end but not at the 3′ end. While sgR16(9–22) and sgR16(1–22) did not reduce the miR-16 level at all, sgR16(1–14) significantly reduced the level to 58% (Figure 4A). These results contrast with those obtained with a transfection reagent, in which sgR16(1–22) almost completely eliminated cellular miR-16 (Figure 1D).

We also carried out a similar experiment with HL60 cells using 5′- and 3′-phosphorylated sgR16(1–14) and sgR16(1–22). Likewise sgR16(1–14) significantly reduced the miR-16 level to 53%, but in contrast sgR16(1–22) also did work almost perfectly (Figure 4B). This was also the case in HEK293 cells (data not shown). This discrepancy would be attributed to the phosphorylation at the 3′ end, which appears to make RNA more efficient in being taken up by human cells and/or more stable in the cells than RNA with no 3′-phosphate judging from a result of the RNA survivability test (data not shown).

Furthermore, to show that the knockdown of miR-16 is functional, we examined its endogenous target, the Bcl-2 mRNA, for the stability [30]. The Bcl-2 mRNA levels in HL60 cells were upregulated to 122% and 137% by downregulating the miR-16 levels by sgR16(1–14) and sgR16(1–22), respectively (Figure 4B,C). This result implies that sgR16(1–14) can stabilize the Bcl-2 mRNA by reducing the miR-16 level and subsequently inhibiting the mRNA transport to P-bodies.

### Downregulation of miRNA Expression Levels by Naked 14-nt sgRNAs

**Figure 5:**
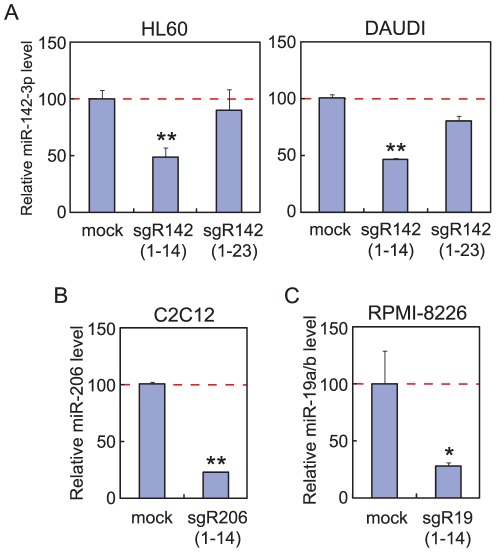
Downregulation of miRNA expression levels by naked 14-nt sgRNAs. (A) Real-time PCR analyses for miR-142-3p were carried out for total RNAs extracted from HL60 and DAUDI cells that were cultured for 24 hours in the absence or presence of sgR142(1–14) or sgR142(1–23). The miR-142-3p levels are normalized against the RNU48 levels. (B) Real-time PCR analysis for miR-206 was performed for total RNA extracted from C2C12 cells that were cultured for 24 hours in the absence or presence of sgR206(1–14). The miR-206 levels are normalized against the sno234 RNA levels. (C) Real-time PCR analysis for miR-19a/b was performed for total RNA extracted from RPMI-8226 cells that were cultured for 24 hours in the absence or presence of sgR19(1–14). The miR-19a/b levels are normalized against the 5S rRNA levels. Error bars indicate s.d. (n = 3). *, P<0.05; **, P<0.001.

We also examined three naked 14-nt sgRNAs against miR-142-3p, miR-206, and miR-19a/b for their guiding ability. sgR142(1–14) significantly reduced the miR-142-3p level to 48% and 47% in HL60 and DAUDI cells, respectively, without any transfection reagents, whereas sgR142(1–23) decreased the level slightly in both cell lines (Figure 5A). The 22-nt 5′- and 3′-phosphorylated sgR16(1–22) worked very efficiently even in a naked form (Figure 4B), whereas the 23-nt sgR142(1–23), which was also 5′- and 3′-phosphorylated, barely worked, suggesting that, unlike 14-nt sgRNAs, the efficiency of naked 22–23-nt RNAs in being taken up by living cells and/or their stability in the cells may change depending on their sequence itself as well as 3′-phosphorylation status.

The miR-206 expression in C2C12 cells was efficiently downregulated to 22% by sgR206(1–14), and the miR-19a/b level in RPMI-8226 cells was reduced to 28% by sgR19(1–14) (Figure 5B,C). These observations imply that in general we can eliminate a specific cellular miRNA at least by ∼50% by using 1 µM of a naked 14-nt sgRNA on the basis of TRUE gene silencing.

### miRNA-targeting RNA Therapy

Our results suggest that TRUE gene silencing can be used for RNA therapy targeting disease-causing miRNA. miRNAs to be targeted would include miR-19a/b and miR-21 for cancer therapy and miR-122 for hypercholesterolemia and hepatitis C treatments. As 14-nt sgRNA can be taken up easily by the cells without any carrier reagents such as cholesterol and cationic liposome, and can guide tRNase Z^L^ to cleave miRNA, TRUE gene silencing may be advantageous over a technology based on the inhibitory mechanism through binding and sequestering [20]–[22].

## Supporting Information

Figure S1 **The reduction in the miR-16 level by sgR16(1–14) is attributable at least partly to tRNase Z^L^.** (A) Quantitation of the miR-16 level in HEK293 cells with a LightCycler 480 SYBR Green I Kit. The HEK293 cells were transfected with mock, 35 nM of sgR16(1–14) or sgR16(1–22) together with mock or the anti-tRNase-Z^L^ siRNA. sgR16(1–14) and sgR16(1–22) were phosphorylated at both 5′ and 3′ ends. The miR-16 levels are normalized against the 5S rRNA levels. Error bars indicate s.d. (n = 3). *, P<0.001. (B) Western blot analysis. The tRNase Z^L^ level under the above transfection conditions was analyzed. β-actin was used as a loading control.
(PDF)

Figure S2 **Naked 14-nt sgRNA can be taken up by living cells.** (A) Confocal laser microscopic images of HeLa cells. HeLa cells were fixed and stained with ethidium bromide 24 hours after the cells were cultured in the absence or presence of 1 µM of the naked 14-nt 3′-FITC-labeled sgRNA14. (B) Fluorescence ratiometric analysis. Fluorescence ratiometric images of HeLa, HEK293, and Jurkat cells were taken 24 hours after the cells were cultured in media containing 1 µM of the 3′-FITC-labeled sgRNA14. (C) sgRNA survivability tests. The 3′-FITC-labeled sgRNA14 was analyzed on a denaturing 20% polyacrylamide gel. The standard 3′-FITC-labeled sgRNA14 (S) and the sgRNA14 retrieved from inside the cells (R) are shown. Asterisks denote the recovered sgRNA. The bands shifted upward would correspond to complexes between the sgRNA and small cellular RNAs.
(PDF)

Text S1 **Supplementary Materials and Methods.**
(PDF)
