# Supplementary figures and images for: Elimination of Specific miRNAs by Naked 14-nt sgRNAs
Source: PLoS One. 2012 Jun 4;7(6):e38496. doi: 10.1371/journal.pone.0038496 (PMC3366928; doi:10.1371/journal.pone.0038496)

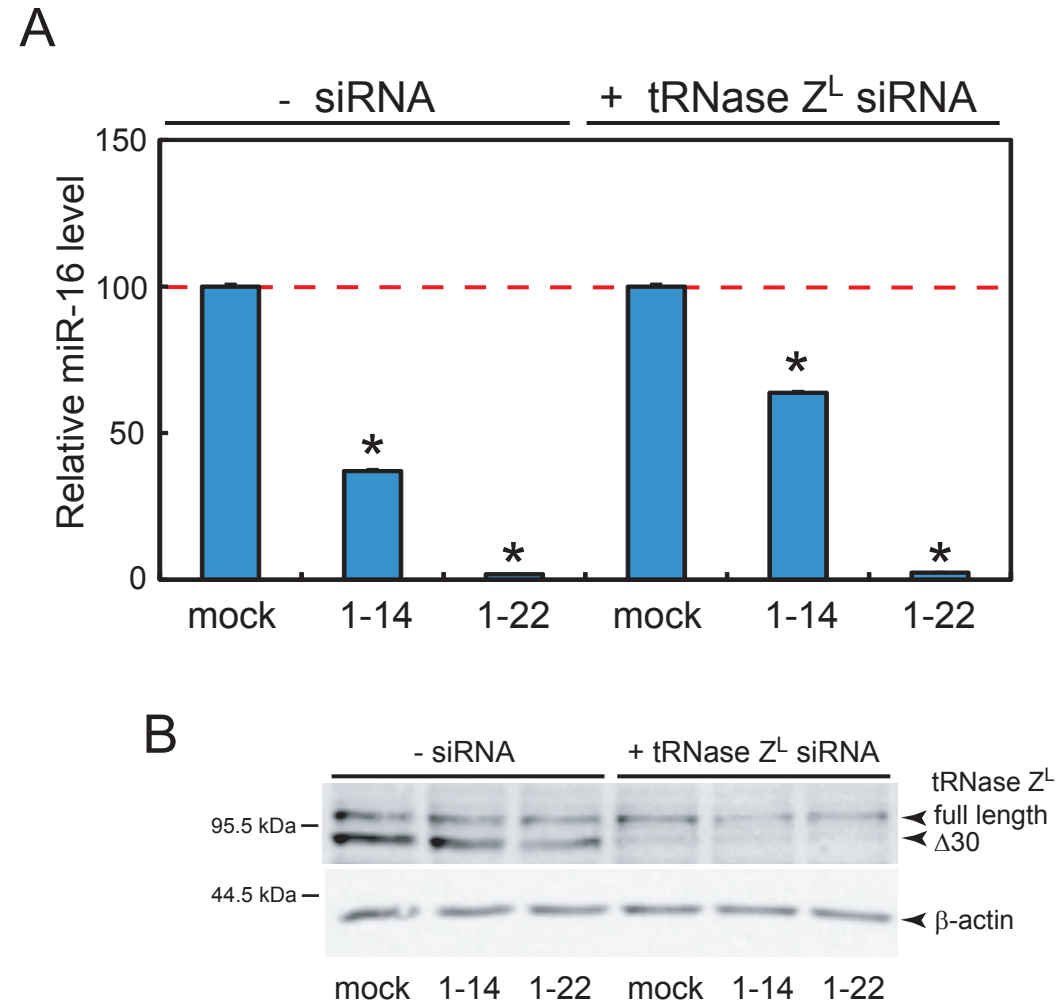

Figure S1

Supplement: Figure S1 — The reduction in the miR-16 level by sgR16(1–14) is attributable at least partly to tRNase ZL. (A) Quantitation of the miR-16 level in HEK293 cells with a LightCycler 480 SYBR Green I Kit. The HEK293 cells were transfected with mock, 35 nM of sgR16(1–14) or sgR16(1–22) together with mock or the anti-tRNase-ZL siRNA. sgR16(1–14) and sgR16(1–22) were phosphorylated at both 5′ and 3′ ends. The miR-16 levels are normalized against the 5S rRNA levels. Error bars indicate s.d. (n = 3). *, P<0.001. (B) Western blot analysis. The tRNase ZL level under the above transfection conditions was analyzed. β-actin was used as a loading control. (PDF) [file pone.0038496.s001.pdf]

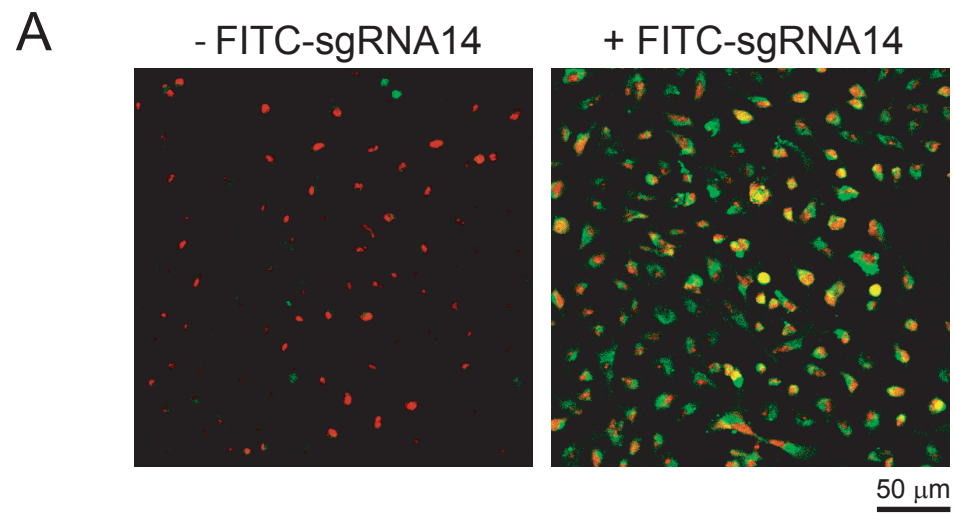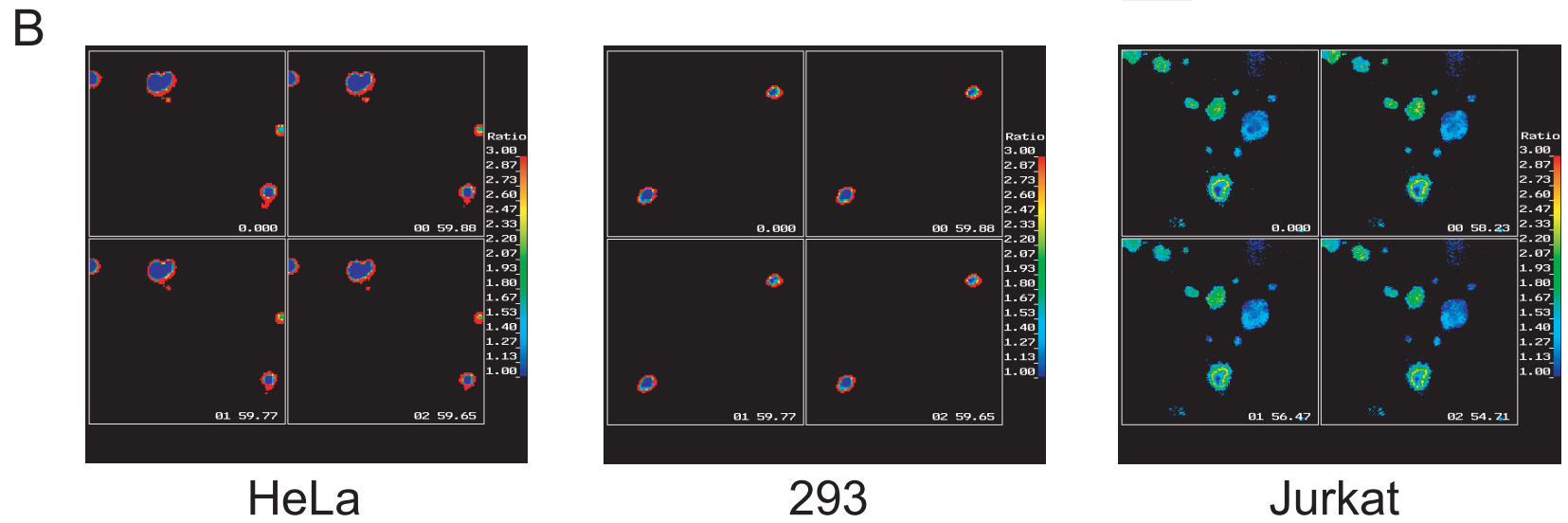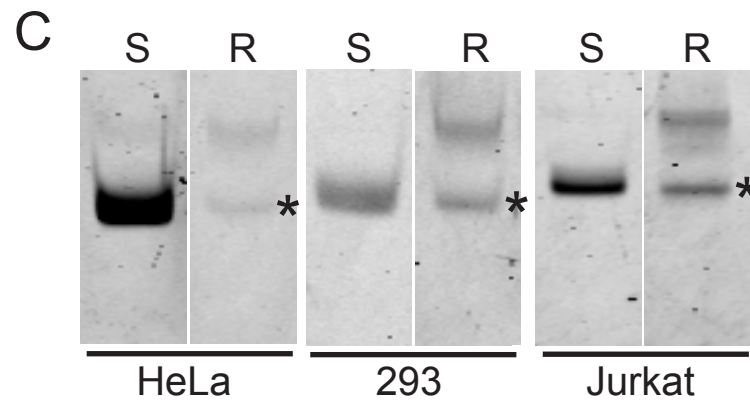

Figure S2

Supplement: Figure S2 — Naked 14-nt sgRNA can be taken up by living cells. (A) Confocal laser microscopic images of HeLa cells. HeLa cells were fixed and stained with ethidium bromide 24 hours after the cells were cultured in the absence or presence of 1 µM of the naked 14-nt 3′-FITC-labeled sgRNA14. (B) Fluorescence ratiometric analysis. Fluorescence ratiometric images of HeLa, HEK293, and Jurkat cells were taken 24 hours after the cells were cultured in media containing 1 µM of the 3′-FITC-labeled sgRNA14. (C) sgRNA survivability tests. The 3′-FITC-labeled sgRNA14 was analyzed on a denaturing 20% polyacrylamide gel. The standard 3′-FITC-labeled sgRNA14 (S) and the sgRNA14 retrieved from inside the cells (R) are shown. Asterisks denote the recovered sgRNA. The bands shifted upward would correspond to complexes between the sgRNA and small cellular RNAs. (PDF) [file pone.0038496.s002.pdf]
