# Supplementary material for: Elimination of Specific miRNAs by Naked 14-nt sgRNAs
Source: PLoS One. 2012 Jun 4;7(6):e38496. doi: 10.1371/journal.pone.0038496 (PMC3366928; doi:10.1371/journal.pone.0038496)
Supplement: Text S1 — Supplementary Materials and Methods. (PDF) [file pone.0038496.s003.pdf]

Supplementary Materials and Methods

Confocal laser microscopy

HeLa cells were incubated in a staining chamber containing 10% FBS and 1 μM of the 14-nt 3′-FITC-labeled sgRNA14. After 24 hours, the cells were fixed, mounted and visualized using a confocal laser scanning microscope MRC-1024 (Bio-Rad). The nuclei were stained with ethidium bromide.

Fluorescence ratiometric imaging

HeLa and HEK293 cells were incubated in ploy-L-lysine-coated glass-bottom microwell dishes containing medium with 10% FBS and 1 μM of the 3′-FITC-labeled sgRNA14. After 24 hours, the cells were rinsed twice with 1 × PBS, and visualized using an inverted epifluorescence microscope (Zeiss) and an ICCD camera (Hamamatsu). Jurkat cells were grown in medium containing 10% FBS and 1 μM of the 3′-FITC-labeled sgRNA14. After 24 hours, the cells were rinsed twice with 1 × PBS, transferred to a poly-L-lysine-coated glass-bottom microwell dish, and visualized likewise.
